# Fingertip tactile sensation via piezoelectric micromachined ultrasonic transducers with an amplified interface

**DOI:** 10.1038/s41598-024-52630-2

**Published:** 2024-02-01

**Authors:** Junji Sone

**Affiliations:** Tokyo Polytechnic University, 5-45-1 Iiyama Minami, Atsugi, Kanagawa 243-0297 Japan

**Keywords:** Engineering, Mechanical engineering

## Abstract

Tactile devices are often used in the field of robotics; however, the development of compact high-resolution tactile devices remains challenging. In this study, we developed a haptic device for force presentation using a DC motor and a tactile sensation device to simultaneously present haptic and tactile stimuli. A microelectromechanical system was selected to maintain the compactness of the tactile device. Piezoelectric micromachined ultrasonic transducers are known for high-power stimulation, and we selected lanthanum-doped lead zirconate titanate as the high-power amplified actuator. A finger mount structure that transfers force for amplifying ultrasonic waves was considered to combine acoustic pressure and aeroacoustics by attaching silicone rubber. The device was fabricated, and the performance of the tactile sensations was evaluated. The developed device uses the novel concept of combining acoustic pressure and aeroacoustics, and its compactness renders it suitable for wearable systems.

## Introduction

A tactile display is used to exhibit the unevenness of the surface of an object touched by a finger, such as the shape of edges and slippage when gripping an object, whereas a haptic display is used to show the reaction force when touching an object and the shape of the object when gripping, mainly presenting reaction forces from objects, gravity, etc. Tactiles use four types of mechanoreceptors in the finger skin: FAI (Meissner corpuscles, sensitivity 20–40 Hz, RA), SAI (Merkel cells), FAII (Pacinian corpuscles, sensitivity 250 Hz, PC), and SAII (Ruffini endings). These mechanoreceptors are located at different depths and perform their own tasks and respond quickly. However, haptic sensation is felt in muscle spindles, Golgi tendon organs, joint receptors, and mechanoreceptors in the skin^1^. Tactile sensation requires a frequency of 250 Hz and an approximately 2 mm density of the two-point discrimination threshold of the finger to stimulate mechanoreceptors^2^.

Several static and dynamic devices have been developed for tactile research^3^. An optical to tactile converter (Optacon, 1.1–2.1-mm pitch, 6 × 24 array, 250 Hz) is a well-known stationary type^4^. Killebrew et al. developed a large, high-performance device with 400 pins in the 38–25 mm range and an operating frequency of 500 Hz^5^. Ikei et al. conducted research using a vibrating pin array tactile display with 11 × 6 pins arranged at a 2 mm pitch, which can present stimuli at 230–700 Hz, achieving high density and high speed^6^. Hudin also developed a device that generates tactile friction on the surface of a thin glass plate^7^. However, these systems are not suitable as wearable systems. Various driving methods, including piezoelectric, SMA, pneumatic, electromagnetic, electrotactile, and motor methods are used; details are summarized in the literature^3^.

Wellman et al. developed a high-bandwidth shape-memory alloy tactile display as a compact device that can present tactile sensations. A total of 10 pins are arranged vertically at 2 mm intervals and the liquid cooling is used for the SMA to achieve 40 Hz operation^8^, however the operating speed is insufficient. VITAL^9^ uses microcoils and magnets to achieve 800 Hz with 8 × 8 pins spaced at 5 mm intervals, but the resolution is low. STReSS^10^ presented a tactile sensation at 700 Hz in a 10 mm square area by bending and deforming 10 × 10 piezoelectric cantilevers. This method uses a cantilever in an upright position, which increases the height of the device. Summers et al. developed a device with 100 pins arranged in a 10 mm × 10 mm area and driven by a piezoelectric bimorph at 20–400 Hz^11^. This study investigated different aspects of spatiotemporal perception mediated by different mechanoreceptor populations and showed that the responses of different receptor populations to stimuli from an array are both individual and interactive. The above is considered to be better. This device is relatively large, making it difficult to use as a wearable device. A tactile display that uses the thermal expansion and contraction of nichrome wire for stimulation has also been developed^12^, with 6 × 24 pins, rows of 1.1 mm, and columns of 2.1 mm density, and can present lateral vibrations of 320 Hz. Although a high wire temperature is needed, pins can be placed at a high density. The tactile pattern and discrimination of motion direction can be distinguished. Expansion to multifinger devices requires consideration of wire placement.

With respect to the development of a multifinger tactile device^13^, Caldwell et al. developed a device with a size of 30 mm × 30 mm × 12 mm and a 4 × 4 pin on a 15 mm × 15 mm square driven by air pressure using a low friction microcylinder^14^. However, the operating frequency is 11 Hz, the presentation density is low, and textures can be displayed. Kim et al. used linear ultrasonic actuators in an 8 × 4 pin array arranged at 2 mm intervals and operated at 20 Hz^15^. The device is compact at 18 × 25.5 × 13.5 mm, but the operating frequency is low and effective for stimulating FAI. Sarakoglou et al. developed a device that uses a DC motor to drive a 4 × 4 (15 mm × 15 mm range) pin array through a flexible tendon^16^. The presentation force is 1–3 N large, the device operates at 12 Hz, and although it is slow, it has little effect on stimulating FAI. Furthermore, the fingertip module size was improved to 32 × 12 × 15 mm, the weight is 30 g, and the frequency was 19 Hz^17^. The Kajimoto group developed an electrotactile display with real-time impedance feedback using pulse-width modulation^18^. The relationship between electrical stimulation and each mechanoreceptor has been investigated^19^, but only the pressure sense has been determined^20^; the relationship with other senses is unclear. As a soft actuator, Koo et al. used an electroactive polymer to create a flexible display with a 5 × 4 array in an 11 mm × 14 mm area, an actuator diameter of 2 mm, and a 3 mm thickness^21^. The operating frequency was 100 Hz, and the displacement was 100 μm. The device structure, which is characteristically soft and flexible, could easily be fabricated. A voltage of 2.5 kV was needed, and the displacement decreased significantly as the frequency increased, making it difficult to drive up to 250 Hz. Lee et al. developed a flexible device using a dielectric elastomer with a 2 × 3 arrangement and a 5 mm spacing between each actuator^22^. The actuator had a large deformation, but the density of the tactile sensation was low, and the frequency was also low at 10 Hz. As described above, tactile displays that present tactile sensations to multiple fingers must consider the variety of sensations they present while satisfying the requirements of compactness, density, and frequency.

Research is underway to simultaneously express high-resolution tactile and haptic stimulation. Chen et al. displayed normal and lateral forces and vibrotactile feedback using a magnetorheological foam actuator, piezoelectric vibrator, and a DC motor^23^. It provides a sense of force and vibrotactile feedback, but insufficient tactile information. Yem et al. developed a device that uses electrical stimulation to present pressure and low-frequency vibrations, shear deformation to the rotation of a motor, and high-frequency vibrations to be presented by a motor^24^. They confirmed that high-frequency vibrations affect friction and fine irregularities; anodal stimulation mainly produces a low-frequency vibration sensation; and cathodal stimulation can produce a rough sensation. Furthermore, we believe that reality will further improve if a force is displayed at the finger joint receptors.

Ultrasonic transducers are the primary components of actuators and sensors. Hosley et al. developed a microelectromechanical system (MEMS) device to read fingerprints using piezoelectric micromachined ultrasonic transducers (pMUTs)^25^; their sensors achieved a high density of 24 × 8 arrays and a pitch of 100 μm. Ultrasound stimulation of the human body using ultrasonic transducers has also been investigated in terms of tactile applications^26^, Iwamoto and Shinoda produced a force at a focal point using a 91-hexagon matrix of transducer arrays with a spatial resolution of 20 mm. They used burst waves ranging from 20 to 250 Hz and small focus points of approximately 70 kHz ultrasound^27^, improving the spatial resolution by 13 mm. A three-dimensional (3D) haptic shape was generated using mid-air-focused ultrasound^28^. Low-frequency lateral modulation with a fine spatial step width was used to realize nonvibratory pressure sensations^29^. However, these studies cannot be applied to multipoint sensations. Lin et al. investigated tactile devices based on aluminum nitride (AlN) pMUTs^30^ and lithium niobate pMUTs^31^. However, these studies did not consider multiple tactile sensations. Another study reported an AlN pMUT-based tactile device^32^, wherein high sensation performance was achieved; however, multiple sensations were not considered. Force and tactile sensations were generated using cable-driven haptic mechanisms and ultrasonic tactile displays^33^. However, further improvements are necessary to achieve the same presentation accuracy for haptic and tactile sensations.

This research employs a piezoelectric micromachined ultrasound transducer actuator to construct a tactile device that has high-density stimulation at a two-point discrimination distance, and a high speed of 250 Hz or more, and is compact enough to be worn on a finger. The proposed tactile display can simultaneously demonstrate the feeling of sliding due to acoustic pressure vibrations and the shapes of edges and other objects generated by aeroacoustics using a single tactile device. Such simultaneous presentation of slip and shape using one device has not been developed before. To transmit force, a finger mount structure (hereinafter referred to as a pad) made of a 3D-printed resin structure coated with metal was used, and a high-density tactile sensation was achieved through low-frequency drive and sound pressure amplification. Silicone rubber is used to create a combined sound pressure and aeroacoustic effect to improve performance. Our project developed a multifinger haptic device and evaluated its performance^34^. The goal is to integrate this approach with a haptic display to simultaneously define haptic and tactile sensations by cosensation with finger mechanoreceptors, muscle spindles, Golgi tendon organs, and joint receptors.

## Finger pad tactile display construction

### Construction of pMUTs

#### Deposition of the piezoelectric film

Lead zirconate titanate (PZT) is widely used for MEMS actuators; in this study, lanthanum-doped PZT (PLZT) was selected owing to its excellent piezoelectric properties^35^. A Pb:La:Zr:Ti (109.3:0.7:52:48) sol–gel solution (N solution 17%; Mitsubishi Materials Corporation) was used. A piezoelectric film with a thickness of approximately 125 nm was fabricated via spin coating (acceleration of 0.5 s) at 4200 rpm for 30 s. These conditions differed from those of the motor torque of the spin coater. An MSB-100 (Mikasa) was used under the aforementioned conditions, and a headway spinner (UCB) was operated at 3000 rpm for 20 s at an acceleration of 1.0 s. Spin coating was performed twice to obtain a stacking thickness of 250 nm. Rapid thermal annealing (RTA; IR-HP2-9, Yonekura Seisakusho Co., Ltd.) was performed at 700 °C for 1 min, and the temperature was increased at a rate of 20 °C/s. The entire film fabrication procedure was repeated to obtain a 2-μm-thick PLZT film.

**Figure 1:**
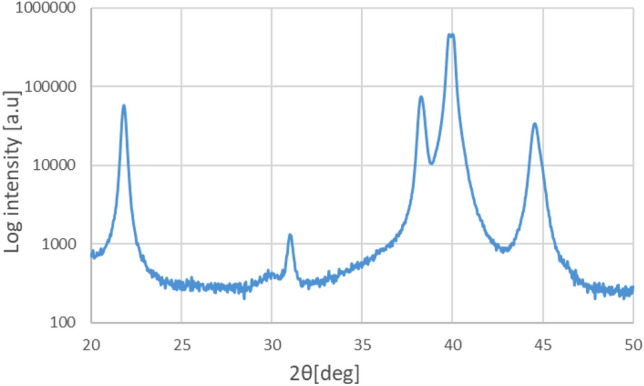
XRD measurement results for the PLZT film.

**Figure 2:**
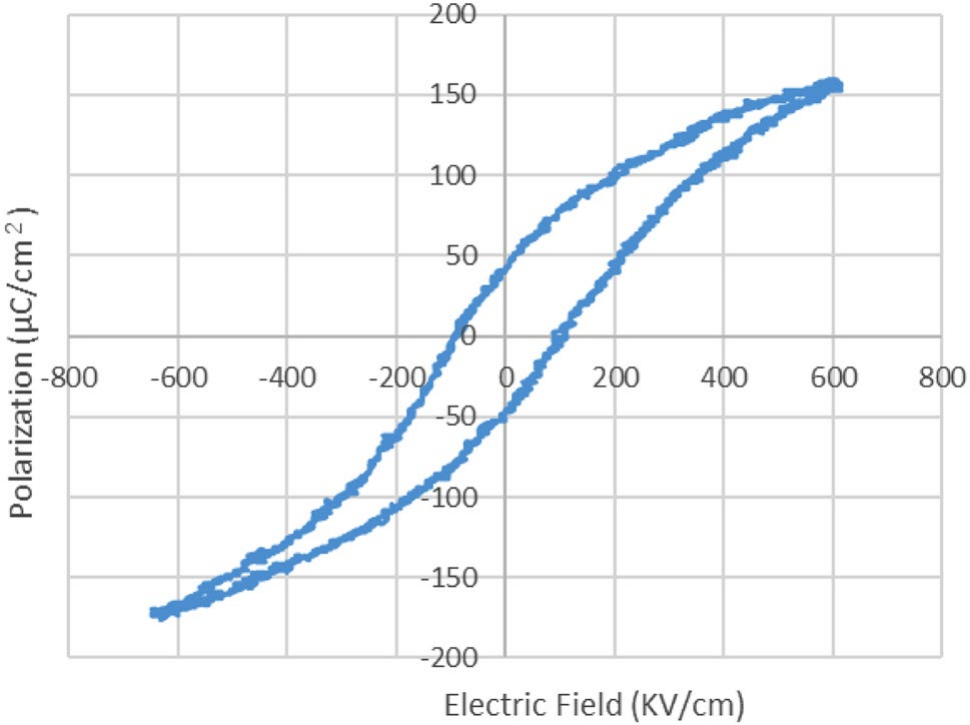
Polarization versus electric field hysteresis measurement results.

Figure 1 depicts the X-ray diffraction (XRD) patterns of the resulting films. The XRD peaks at 22° (PZT 100) and 38° (PZT 111) correspond to primary PLZT crystals. Figure 2 illustrates the film polarization with respect to the electric field hysteresis. These data were obtained by applying 50 Hz ± 15 V triangle waves using a signal generator (AFG310, Tektronix) and a piezo amplifier (M-2682, MESS-TEK Co., Ltd.). The input voltage and Sawyer–Tower circuit voltage (C = 0.1 μF) were plotted, with the remanent polarization (Pr) set at 50 μC/cm^2^ and coercive field (Ec) set at 100 kV/cm. Typically, a smaller coercive electric field (narrow hysteresis) is better for vibration generation^36^. The piezoelectric constant *d*_31_ of the film was − 218 pC/N at a cantilever thickness of 80 μm, which was determined using a laser Doppler vibrometer (UHF-120, Polytec Inc.). Equation (1) in^37^ was used to calculate *d*_31_ using the measured cantilever thickness, film thickness, and deformation displacement. According to the provider of the PLZT sol–gel solution (Mitsubishi Materials), these piezoelectric constant results were caused by a stiffness reduction in the piezoelectric film with the addition of lanthanum. The cantilever significantly constrained the vibration of the piezoelectric film compared to that of the bulk piezoelectric material.

#### Device fabrication

**Figure 3:**
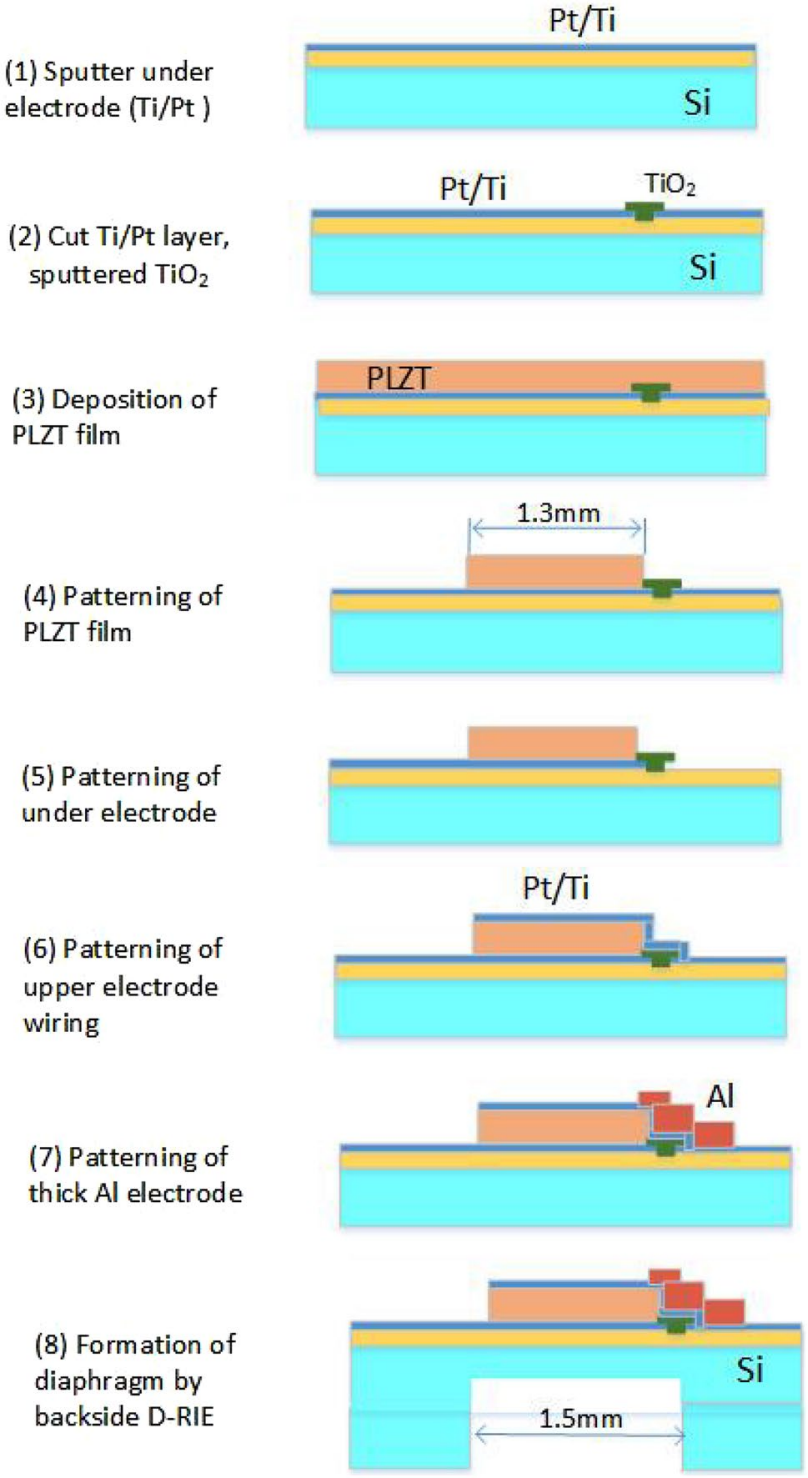
Schematic illustrating the device fabrication process.

**Figure 4:**
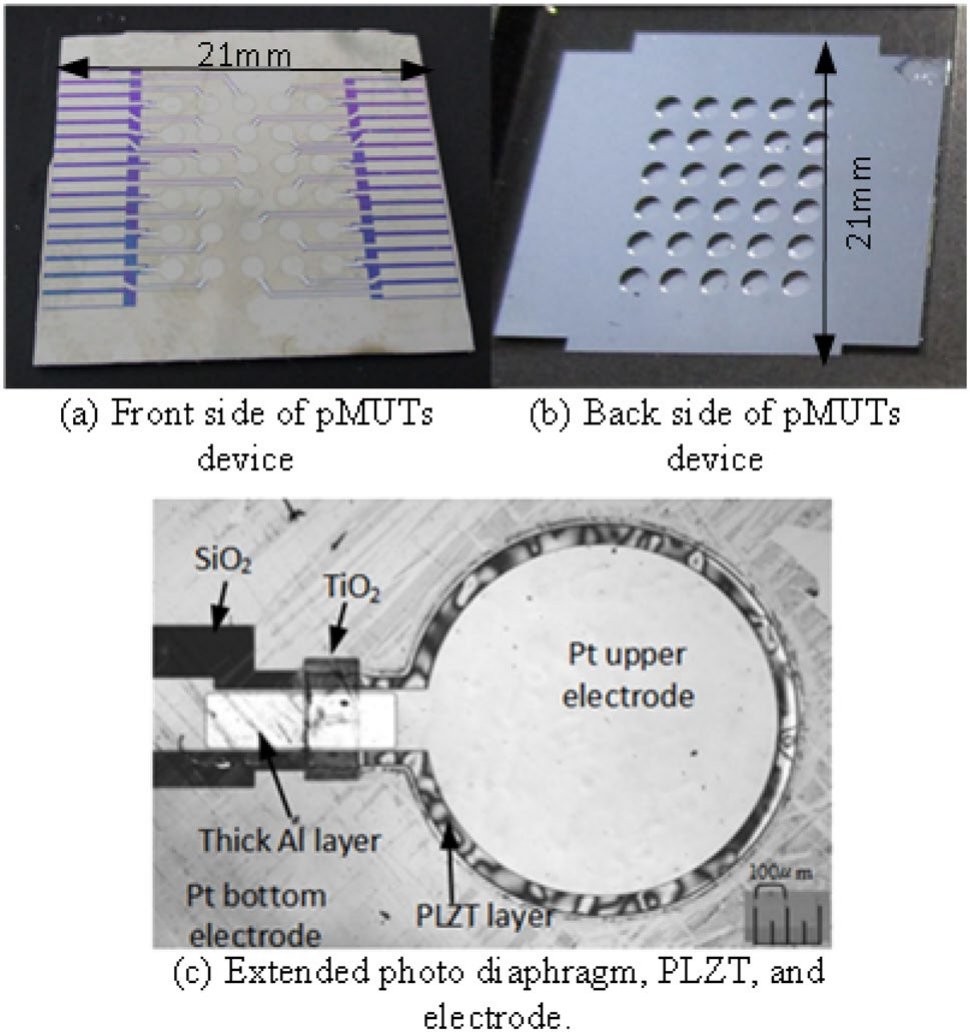
Fabrication results.

Figure 3 shows a schematic of the device fabrication process. In Step (1), a 20-nm-Ti/200-nm-Pt bottom electrode is sputtered on a 500-nm-SiO_2_/Si wafer. In Step (2), a small region (50 μm) of the Ti/Pt layers is cut via ion milling for separate ground and voltage supplies connect wires. Additionally, a 40-nm-thick TiO_2_ layer is deposited on the SiO_2_ layer to prevent the generation of lead compounds, which would have compromised insulation^38^. In Step (3), a 2-μm-thick PLZT layer is deposited by sixteen rounds of spin coating and eight rounds of RTA, with one RTA round performed for every two rounds of spin coating. To increase the vibration strength, it is necessary to increase the film thickness, but we decided to use a 2 μm thick film because of the stability of the sol–gel film formation. The spin-coating area was expanded to more than twice the area of the device, and areas with unstable film thickness were not used. In Step (4), the PLZT film is patterned using the HBF_4_:H_2_O (1:10) solution during wet etching^39^ for 1–2 min while observing the residual amount of the PLZT film. In Step (5), the Ti/Pt bottom electrode is patterned via ion milling. In Step (6), a 20-nm-Ti/200-nm-Pt upper electrode is sputtered and patterned using a lift-off process, and in Step (7), a 1.2-mm-thick Al layer is evaporated between the 2-μm-thick PLZT and insulator layers. In Step (8), the diaphragm is formed by deep reactive ion etching of the back side of the device (MUC-21, Sumitomo Precision Products Co., Ltd.). Therefore, the actuator has a Si diaphragm with a thickness of 55–75 μm, measured using a laser microscope (Lasertec Optelics Hybrid LS-SD), a 500-nm-thick SiO_2_ layer, a 20-nm-Ti/200-nm-Pt bottom electrode, a 2-μm-thick PLZT layer, and a 20-nm-Ti/200-nm-Pt upper electrode. An actuator array (diameter = 1.5 mm) is placed in an area of 9 mm × 11 mm. Figure 4 illustrates the fabrication results. The actuators are mostly aligned at two-point discrimination distances. Figure 4c shows the piezoelectric film, the upper electrode on the diaphragm, and the 1.2-μm-thick Al layer covering the wiring on the PLZT layer. Here, the pMUTs are 1.5 mm in diameter, and the Si diaphragm is 55–75 μm thick. The diameter and thickness of the PLZT piezoelectric actuator are 1.3 mm and 2 μm, respectively.

### Constructing the finger pad

**Figure 5:**
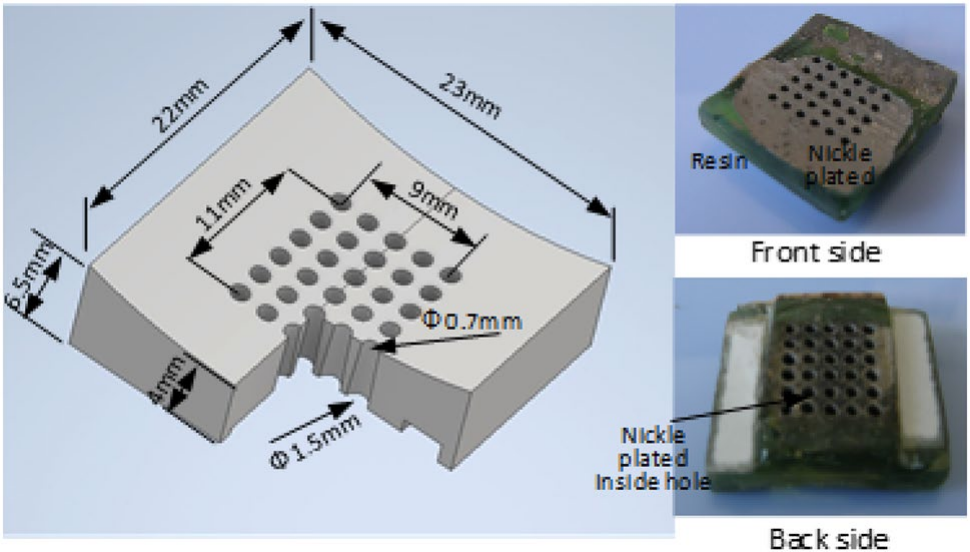
3D CAD model of the finger pad (Cut to 1/4 and displaying the cross section). Finger pad design and electroplating results.

The finger pad, comprising ultrasonic amplified tubes, is the primary device for tactile sensation and can be used for haptic sensation (force presentation). Figure 5 shows a 3D image and the results of the fabricated finger pad. The body was built using a 3D printer (NOVA3D Elfin2), and photopolymerization-based 3D printing was used to obtain a high-precision shape. The inner shape was tapered, and the surfaces of the tubes were coated with a 120-μm-thick nickel electroplated coating, with 20-μm-thick electroless nickel plating (Japan Kanizen pink [sensitizer]-and-blue solution) and 100-μm-thick electric nickel plating performed using the sulfamate method. Hard metals such as tungsten, cobalt, and titanium can be used, but they are difficult to electroplate, so nickel was chosen for fabrication.

### Simulation of the device

Acoustic wave transfer simulations indicated that a hard material could reflect an ultrasonic wave, generating a power concentration of waves. The horn-like shape reduced the resonance frequency. An axisymmetric simulation model was constructed, and the simulations were performed using COMSOL Multiphysics (6.0, 6.2 : COMSOL was only updated during the research period, and there was no impact on the functions used); specifically, solid mechanics, electrostatics, pressure acoustics, and piezoelectric effect modules were used. A normal PZT (*d*_31_ =  − 40 pC/N estimated from *ɛ*_31_) was used for the simulation, with a drive voltage of 18 V. The entire acoustic pressure of the model was computed by simulation for each step by changing the small step frequency (20 Hz step) and selecting the pressure values at the resonance point. If the resolution of the resonance point was insufficient, the peak area was calculated in 10 Hz increments. During the analysis, the pad was assumed to be attached to the finger (epidermis and bone layer). However, the finger had a fingerprint, and the pad surface could not touch the entire surface of the finger. Therefore, the epidermis and bone layers were removed, and the acoustic pressure at the top point of the pad was evaluated.

Initially, the performance of a single diaphragm was considered. Table S1 lists the acoustic velocities of these materials. Figure S1 depicts the open-case model (one diaphragm, axisymmetric), where the evaluation point is the 4 mm upper point of the vibrator. Figure S2a shows the acoustic simulation results in the range of 20 000–60 000 Hz considering an 80-μm-thick diaphragm; four resonance peaks are observed. Figure S2b depicts the acoustic pressure measurement results. In Figure S2a and b, the peak frequencies at higher frequencies differ because of the wired structure of the MEMS device, wherein the PLZT and electrode are extended for the wired part; however, the base resonance at approximately 22 kHz remains almost the same. Three-dimensional multiphysics simulation is extremely difficult owing to limited computer resources. Therefore, a 2D model with a wiring thinner was created. Figure S4 shows the irregular-shape displacement simulation results of the frequency scan. The model was simplified to two dimensions, and a half thick piece of wire (piezo-electric, 1 μm) was added to the left side, and a 30 V actuator and wire were added. The addition of a wire produced another resonance. The actual model is more complex, but the resonance frequency over the second changes, as shown by the blue circle (when wires added, the peak position shifts. However, since the graph is plotted at the same location, the peak value has decreased.). Figure S3 shows the simulation results of a 50-μm-thick diaphragm, wherein the resonant peaks are slightly greater than those observed for the 80-μm-thick diaphragm. If the diaphragm is thick, it will resist vibrations; however, if it is thin, its resistance to vibrations will decrease. Therefore, the resonance peak value of the 50 μm diaphragm is slightly greater than that observed for the 80 μm thick diaphragm.

Subsequently, the performance of the metal horn pad was considered. Figure S5a depicts the corresponding analytical model. The evaluation was performed at the exit point of the pad, and the diameter of the open hole at the top of the horn was 0.4 mm. Figure S7 shows the acoustic simulation results in the range of 10 000 to 60 000 Hz for an 80-μm-thick diaphragm. The width of the simulation model was expanded from 15 to 70 mm for comparison with Figures S2a and S7. In comparison with Figure S2a, the resonance frequencies were reduced by approximately 10 000 Hz when the pad was used. The resonance frequency was assumed to decrease from 24 to 11 kHz and from 41 to 34 kHz. As indicated in Figure S6, the location of the diaphragm (upside and downside) was determined by considering that the outside of the pad was covered with nickel, which shortend the wiring of the MEMS chip. Figure S9 shows a comparison of the acoustic pressures considering the upward and downward positions of the diaphragm. Although placing the diaphragm downward may deteriorate the performance, the downward diaphragm was selected for this study to avoid short wiring. Figure S10 depicts the acoustic pressure of the nickel horn pad, with the diaphragm thickness varying between 30 and 80 μm. Figure S11 shows the acoustic pressure of the nickel horn pad covered with silicone rubber. The simulation model is shown in Figure S5b, and the upper surface of the silicone rubber is considered as the point of evaluation. A thinner diaphragm (30 μm) reduces its resistance to deformation and increases the amount of vibration. However, a diaphragm thickness of 30 μm results in the best performance; however, the residual stress in the PLZT may damage a thin diaphragm.

**Figure 6:**
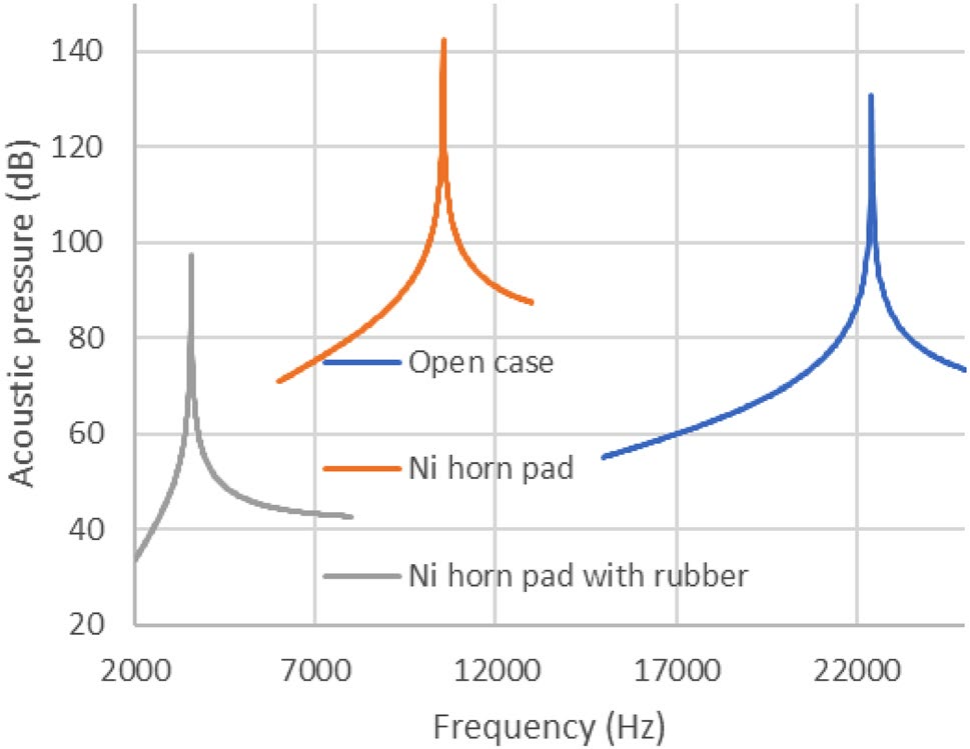
Acoustic pressure simulation results of the three types of devices. (Open, Ni horn pad, Ni horn pad with rubber, 50 μm-thick diaphragm).

**Figure 7:**
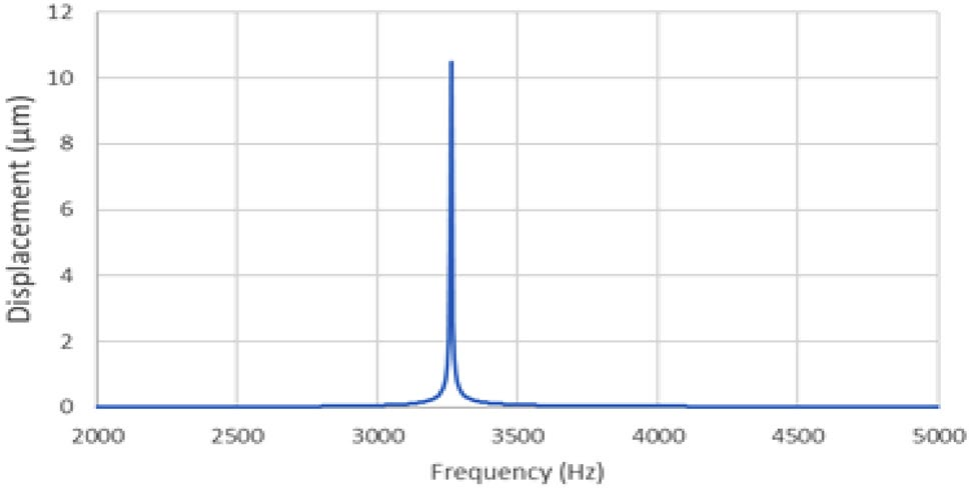
Surface displacement simulation result of silicone rubber calculated by coupling acoustic pressure and aeroacoustic.

Figure 6 depicts the resonance simulation results for the open-case model, considering the nickel horn finger pad with and without silicone rubber. The amplified horns reduced the resonance frequency by 1/2.5, whereas the silicone rubber reduced the resonance frequency by 1/10 in the open-case model (without the pad). In general, silicone rubber allows ultrasonic waves to pass through and can be subjected to aeroacoustic vibrations. Figure 7 depicts the surface displacement of silicone rubber calculated using coupling aeroacoustics (linearized Euler equations) and pressure acoustics, with an applied voltage of 0–30 V and a maximum vibration of more than 10.5 μm. which can stimulate receptors in human fingers and increase the applied voltage 0–48 V, the maximum vibration increased more than 16.8 μm, (80 μm diaphragm case).

**Figure 8:**
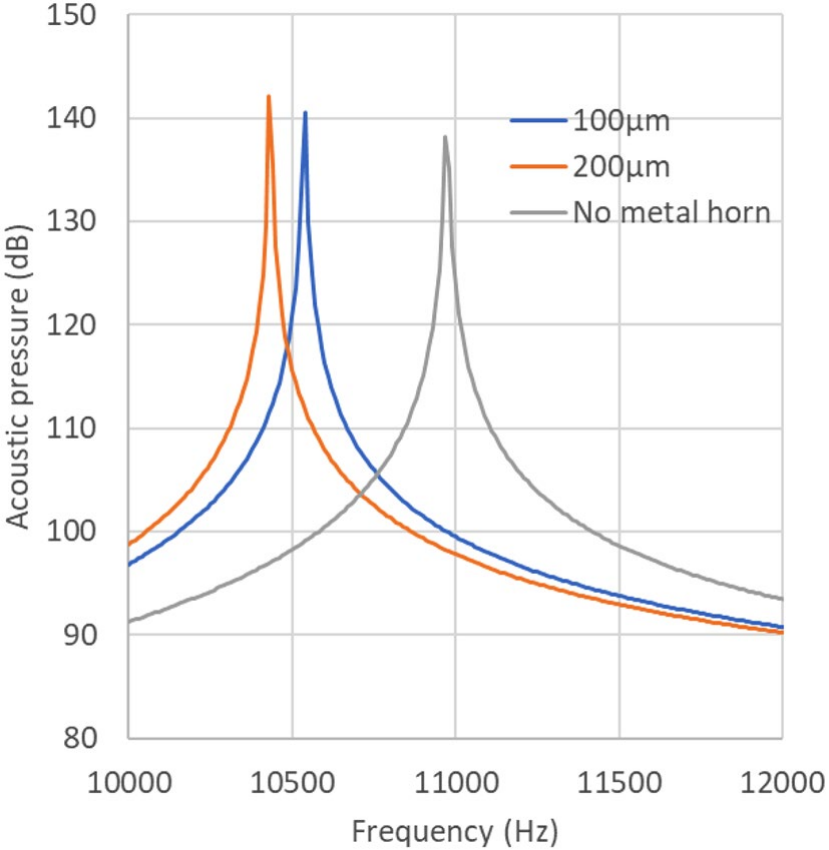
Acoustic pressure simulation results by changing the electroplate thickness.

Figure 8 shows the acoustic pressure simulation results with and without the nickel horn. As indicated in the figure, the presence of a nickel horn improves the acoustic pressure and reduces the resonance frequency.

## Performance measurement results

### Acoustic pressure measurement

**Figure 9:**
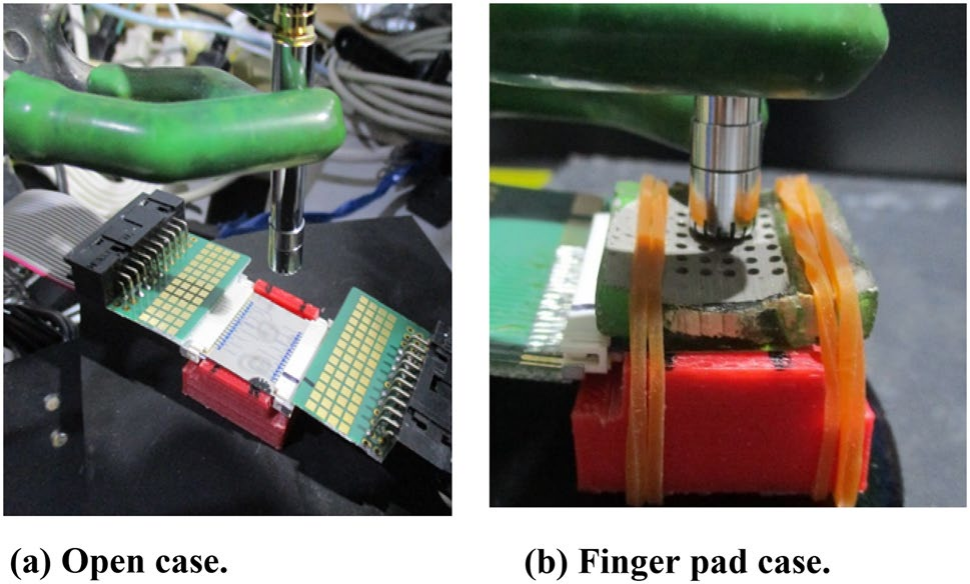
Acoustic pressure measurement method.

**Figure 10:**
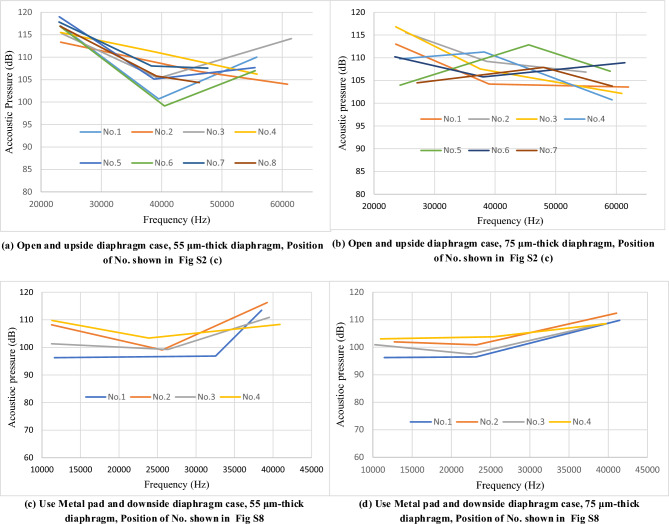
Acoustic pressure resonance peak measurement results for open case and metal pad case.

The acoustic pressure was measured at the 4 mm upper point of the vibrator for the open-case model (no finger pad; Fig. 9a) and at the 1 mm upper point of the amplified tube pad (Fig. 9b) using a 1/4-inch industrial microphone (ACO 4156N, 20 Hz–80 kHz); sound pressure values above 10 kHz were compensated to account for the decline in measurement sensitivity. A unipolar sine wave in the range of 0–18 V, a function generator (AFG310, Tektronix), and a piezoelectric amplifier (M-2682, MESS-TEK Co., Ltd.) were used. Figures 10a and b depict the three resonant acoustic pressure peaks measured from actual pMUTs, driven by a sine wave of 0–18 V and comprising diaphragms with thicknesses of 55 and 75 μm, respectively; Figure S2c illustrates the actuator positions. The peaks of the 55-μm-thick diaphragm are higher than those of the 75-μm-thick diaphragm. The second and third peaks differ because of the different wiring geometries of each pMUT. The peak values change between Figs. 10a and S2a and between Figs. 10b and S3. This variation is due to differences in the directions of the individual wires and slight changes in the wire width. In addition, although the diaphragm is constructed in a trench from the back, there are slight variations in the thickness and shape of the diaphragm, which affects the peak frequency of vibration. Figure 10c and d depict the three resonant acoustic pressure peaks obtained from the measurement of actual pMUTs with a metal horn pad (Figure S8 illustrates the actuator positions), driven by a sine wave of 0–18 V (downward diaphragm), the acoustic pressure peaks of the first to third resonances should have increased by 10 dB (from Fig. 6), but they only increased by 3–5 dB compared with those of the open case (compared with Figure S2 b). The measurement accuracy is low because of the microphone settings. The measurements were obtained at a distance of 2–3 mm from the horn exit owing to the microphone cover, which obstructed the measurement. In comparison with Figure S7, the frequency resonance observed was nearly the same at 11 000 Hz, and the higher order resonances shifted from 33 000 to 25 000 Hz and from 51 000 to 40 000 Hz from the simulation to the actual device. The reason for this difference is that in the actual device, the diaphragms are adjacent to each other at a pitch of 2 mm, however, in the analytical model, there is only one diaphragm, and the conditions are different.

### Psychophysical evaluation

**Figure 11:**
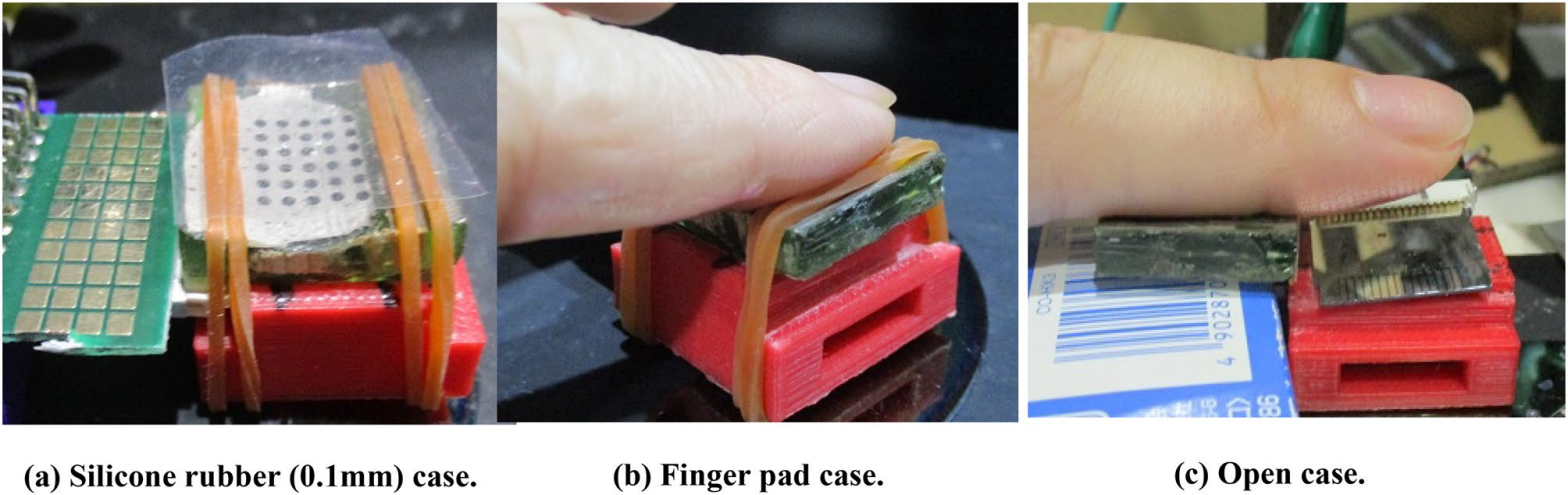
Psychophysical evaluation setting of each evaluation.

**Figure 12:**
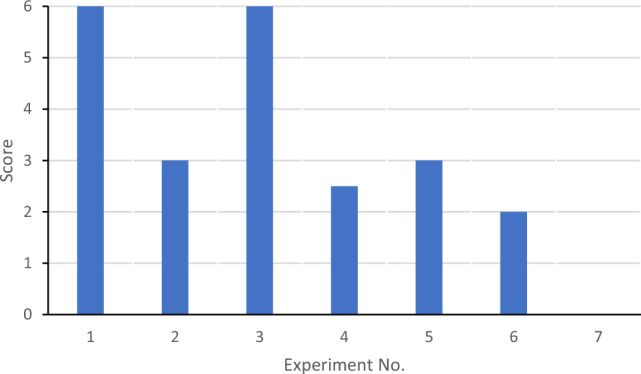
Results of psychophysical evaluation with test subjects, Summation score of psychophysical evaluation for six participants. (Score of each person as 1.0: good, 0.5: weak, 0.0: no recognition).

Five males and one female, aged between 21 and 62 years, participated in nine psychophysical evaluations; Table 1 summarizes the conditions of the experiment. For finger pads covered with silicone rubber, the frequencies were scanned and the highest frequency was used for the experimental analysis. Although Fig. 6 indicates that the resonance frequency is nearly 3600 Hz, a higher frequency is required for adequate performance. Therefore, we used simulations to examine the space when placing the MEMS device and metal horn pad. Figure S12 depicts an analysis model with certain spaces between the MEMS device and the metal horn pad covered with silicone rubber. Figure S13 shows the resonance frequency for each space; the resonance frequency shifts with space. In this experiment, the MEMS device and metal horn pad were attached in place using a rubber band; however, the roughness of the electroplated surface rendered its fixation difficult. The silicone rubber was not fixed and was held in place using only finger pressure. Other experimental frequencies were determined from Fig. 10. In each case, a 200 Hz burst wave (RIGOL DG2052) and a fundamental sine wave of each frequency were used for the experiment (Table 1). No.1 to No.3 are the finger pads with silicone rubber, and No.4 to No.7 are the first resonant frequency experiments for each case. A score of 1.0 implied appropriate recognition of vibration, 0.5 indicated weak recognition, and 0.0 implied no recognition. The evaluations of all participants were collated. Figure 11 presents the images of the psychophysical evaluations of each case, and Fig. 12 illustrates the corresponding results. The points in the figure show the summation of the scores of the six people. (one score of 1.0: good, 0.5: weak, 0.0: no recognition). No.1 to No.3 shows that during the experiment, a 55-μm-thick diaphragm device was completely driven by the application of 0–39 V, whereas a 75-μm-thick diaphragm device could not be entirely driven by 0–48 V and required the application of 0–60 V. Nos. 1, 2, and 3 use the effects of horn-amplified acoustic pressure and aeroacoustic vibration, and this method provides the most vibration of mechanoreceptors. A thick diaphragm has less power for acoustic pressure and aeroacoustic vibration; therefore, 60 V is required for the same sensation level as No. 1. Nos. 4 and 5 use the effect of horn-amplified acoustic pressure; the vibration power is reduced, and scores are almost half of those of nos. 1 and 3. Nos. 6 and 7 are open cases, the acoustic pressure spreads 180 degrees, and these scores are the worst. Aeroacoustic is thought to increase tactile sensitivity because silicone rubber, which is denser than air, vibrates directly on the skin. Several subjects successfully detected low-frequency vibrations from the device with only the pad, whereas a few participants felt sharper vibrations in the case of the silicone rubber-covered device. Therefore, the thickness and material of the silicone rubber should be prioritized. The measurement acoustic pressure of the burst wave of one pMUTs was 124 dB for case no. 6 and 112 dB for case No. 4; the measurement accuracy of No. 4 case was low owing to the microphone settings.

**Table 1:** Conditions of psychophysical evaluation.

Experiment no.	Type of display and experiment method	Diaphragm thickness	Drive signal voltage	Base frequency of200Hz burst wave	Expect acoustic pressure AP) and aeroacousitc displacement (AD)
No.1	Finger pad with silicon rubber (Downside of diaphragm) Recognition of two point vibration on rubber (6.1 mm distance)	55 μm	0–39 V	7550 Hz sin wave	AP: 96 dB, AD:26μmNo
No.2	Finger pad with silicon rubber (Downside of diaphragm)Recognition of two point vibration on rubber(3.5 mm distance)	75μm	0–48 V	8100 Hz sin wave	AP: 93 dB, AD:19μmNo
No.3	Finger pad with silicon rubber (Downside of diaphragm) Recognition of one vibration on rubber	75μm	0–60 V	4900 Hz sin wave	AP: 97 dB, AD:27μmNo
No.4	Finger pad (No silicon rubber, Downside of diaphragm)Recognition of one vibration on pad	55 μm	0–39 V	11240 Hz sin wave	AP: 140dBNo
No.5	Finger pad (No silicon rubber, Downside of diaphragm)Recognition of one vibration on pad	75 μm	0–48 V	12730 Hz sin wave	AP: 138dBNo
No.6	Open (no finger pad and silicon rubber)Recognition of one vibration at 4 mm up point	55 μm	0–39 V	22960 Hz sin wave	AP: 125dBNo
No.7	Open (no finger pad and silicon rubber) Recognition of one vibration at 4 mm up point	75 μm	0–48 V	23610 Hz sin wave	AP: 121 dB

## Conclusions

In this study, a tactile device using pMUTs with a metal horn pad was developed, and the occurrence of tactile sensations was confirmed via a burst wave of 200 Hz using a PLZT piezoelectric film. This horn reduced the resonance frequency of the tactile sensation. In addition, the performance of the device was improved by interfacing the metal horn pad and finger with silicone rubber to produce co-tactile sensations with acoustic pressure and aeroacoustic rubber vibrations. This approach a low-frequency interface, enhancing tactile sensation during psychophysical experiments.

The combination of acoustic pressure and aeroacoustics in a tactile device is a novel concept. In previous studies, Sedat^30^ used an array of 144 pMUTs to induce a sound pressure of 153 dB over 36 mm^2^ (6 mm × 6 mm), Billen^32^ employed 400 pMUTs to achieve 143 dB over 81 mm^2^ (9 mm × 9 mm), and Yue^31^ utilized 24 pMUTs to achieve 133 dB over 35 mm^2^. In these studies, tactile sensations were effectively induced using multiple stimuli^33^. However, the resolution of precise stimulation was not achieved by two-point discrimination. In this study, an acoustic pressure of 124 dB was achieved using one pMUT (39 V drive), leading to a focused stimulation of approximately 0.16 mm^2^. The input voltage (39–60 V; unipolar) was insufficient to stimulate a human finger sensor cell using only acoustic pressure stimulation. However, the combination of acoustic pressure and aeroacoustic rubber vibrations provids sufficient stimulation for finger receptors. A 55-μm-thick diaphragm device realized a practical drive voltage of 39 V. Herein, the resolution of the pMUTs of a high-performance PLZT film achieved two-point discrimination. However, further improvements in aeroacoustic vibrations are required to increase sensory variations.

Previously, electrical stimulation has been proven to be compact, exhibiting sufficient resolution. However, this is only the pressure sense and the sensitivity of the test varies depending on the person as high stimulation causes pain. Given that the nichrome wire tactile device is based on the heating and cooling of a nichrome wire, an extensive cooling system is needed, indicating that obtaining compact devices may be difficult. Flexible polymer devices also stimulate a low-frequency vibration sensation. Additionally, another flexible polymer device with low voltage drive reported by Zhong^40^ cannot achieve high resolution for two-point discrimination of the finger.

The performance of the PLZT piezoelectric films considered in this study was important for building compact devices. Additionally, the wiring method of the MEMS device will improve when direct wire bonding is used, and a compact tactile device will be developed (Figure S14). The tactile device provided finger support for a haptic sensation system with multiple fingers. The developed compact device can be worn and corepresent haptic (force) and tactile sensations. Moreover, a low-voltage drive should be considered to optimize the PLZT film thickness, diaphragm thickness and diameter, and pad structure (horn shape, metal thickness, and materials). In order to respond to changes in posture during haptic sensation, it is necessary to consider how to fix silicone rubber to the metal horn pad. The material and thickness of the film should also be optimized to achieve a sharp tactile sensation. For tactile sensations, consider ways to express small shapes, pressure, slip sensations, etc. Currently, there is only one gradation; therefore, to refine the presentation, we would like to expand it to multitone intensity and consider sensory scales using the JND and limit methods^41^. Furthermore, we consider the simultaneous presentation of tactile and force senses to coordinate with haptic displays. In the future, the device must be further optimized for applications in metaverse and extended reality communication. This approach can also be beneficial for human and robotic communication systems.

## Experiment

This study was approved by the ethics committee of Tokyo Polytechnic University (No. 2023-06) and conducted according to the principles of the WMA Declaration of Helsinki. All participants provided written informed consent prior to the experiments.

### Supplementary Information

Supplementary Information.

## Data Availability

All the data generated or analyzed during this study are included in this published article and its supplementary information file.
