# Supplementary material for: Fingertip tactile sensation via piezoelectric micromachined ultrasonic transducers with an amplified interface
Source: Sci Rep. 2024 Feb 1;14:2629. doi: 10.1038/s41598-024-52630-2 (PMC10831112; doi:10.1038/s41598-024-52630-2)
Supplement: Supplementary file 1 — Supplementary Information. [file 41598_2024_52630_MOESM1_ESM.docx]

**Supplement material**

Fingertip Tactile Sensation via Piezoelectric Micromachined Ultrasonic Transducers with an Amplified Interface

Various simulations of acoustic pressure are executed by COMSOL Multiphysics software (6.0-6.2). Conditions of acoustic velocity are described in Table S1.

Table S1. Acoustic velocity of each material.

1. Open case

Figure S1 shows the analysis model of the open case. This model is axisymmetric. The acoustic pressure measurement point is 4mm upper on the diaphragm. Figure S2 shows resonant frequency (Diaphragm thickness is 80 μm (simulation), 75 μm (Device), driven by 0-18 V sin wave). We observed four resonances with 24, 41, 43, 56KHz. Figure S3 shows the 50 μm thickness of the diaphragm simulation result. Almost the same resonance is observed in the figure S2.

　　

**Figure S1. Analysis model of open case.**


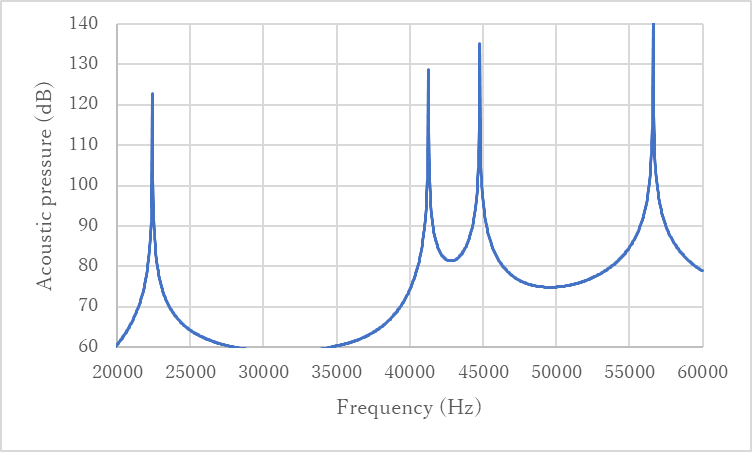


1. **Comsol simulation result (80μm thickness of diaphragm).**


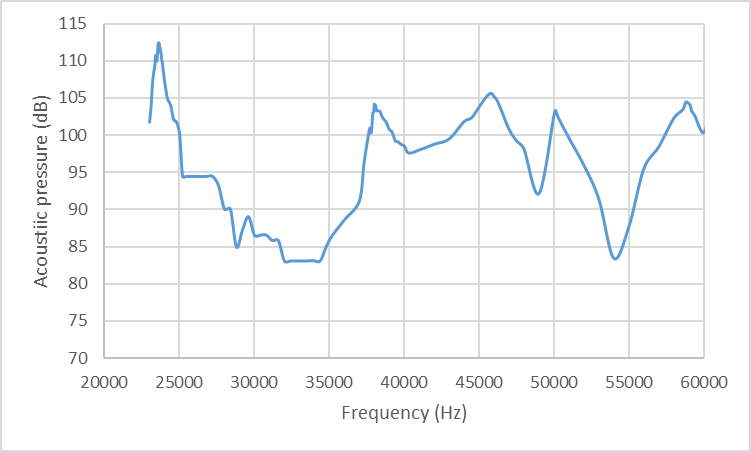


1. **Measurement results (No.1 actuator). (c) Indicates actuator position number.**

**(75μm thickness of diaphragm) (75μm thickness of diaphragm)**

**Figure S2. Acoustic pressure for an open case. (Simulation: 80μm thickness of diaphragm, Devise: 75μm thickness of diaphragm).**


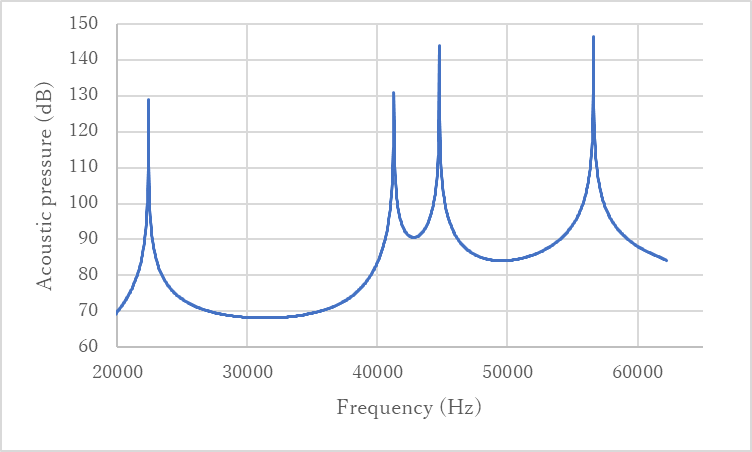


**Figure S3. Acoustic pressure for open case (Simulation, 50 μm thickness of diaphragm).**

**Figure S4. Trial resonance analysis when added with wiring.**

**(Simplified to two dimensional)**

Figure S4 shows the irregular shape displacement simulation result of frequency scan. Model is simplified to two dimensional and half thickness of wire (piezo-electric, 1μm) at left side was added and 30V added actuator and wire. If wire added, another 2nd resonance is appeared. Actual model is more complex, but resonance frequency over 2nd is changing as shown at blue circle (In the case of wire addition, the peak position slid, but the graph was plotted at the same position, and in the case of wire addition, the peak value decreased).

1. Metal horn amplified case

The scheme of the horn model is shown in Figure S5 (a) and Figure S6 indicates that the diaphragm direction is set to upside and downside. Diaphragm thickness is 50-80 μm for simulation. Figure S5(b) shows the horn and silicone rubber.

 　

1. **Metal pad case. (b) Metal pad with silicone rubber.**

**Figure S5 Analysis model with amplified horn.** **Figure S6. Place of Si-diaphragm.**


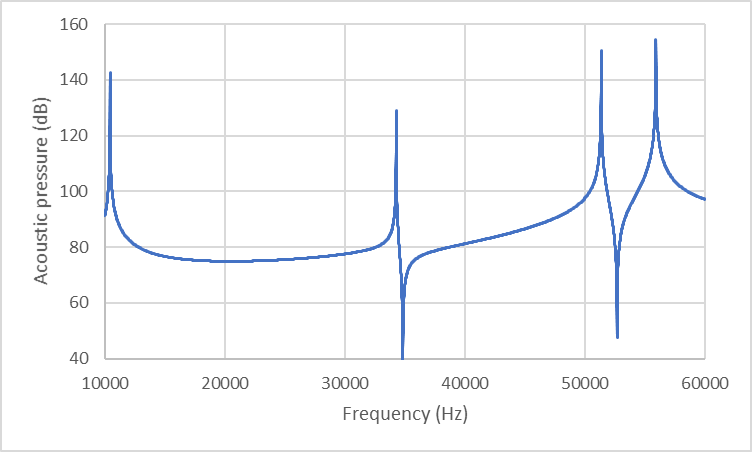


**Figure S7. Wide frequency range simulation of finger metal pad.**

Figure S7 shows the wide-range simulation result of acoustic pressure of a finger metal pad, driven by 0-18V sin wave (The width of the analysis model is expanded from 1.5mm to 7mm), Compared with figure S2 (a), resonance frequencies are almost dropped by about 10000 Hz using the finger metal pad, reduction is inferred (24 -> 11 KHz, 41-> 34 KHz).

**Figure S8. Position numbering of actuator.**

Figure S8 shows the measurement point of acoustic pressure (75μm diaphragm).


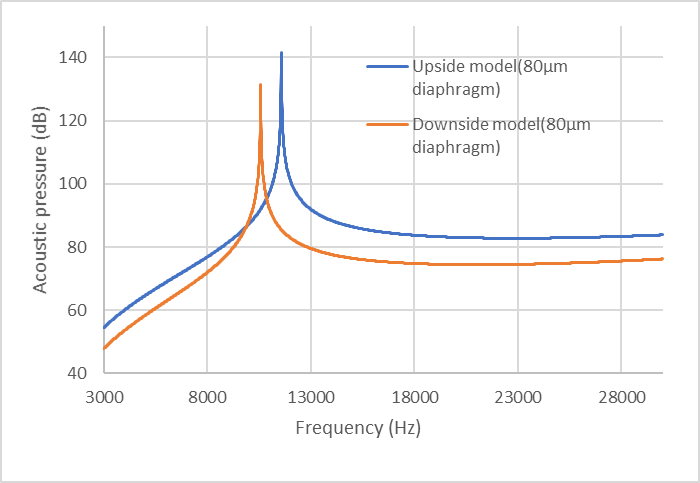


**Figure S9. Acoustic pressure simulation result with metal horn pad for up and down diaphragm.**

Figure S9 shows the comparison of acoustic pressure for up and down places of the diaphragm, driven by 0-18V sin wave. There is a fear of short-circuiting or damage to the wiring and piezo-electric layer because it is installed in an upside case. But the downside case reduces sound pressure by nearly 7dB compared to the upside case from simulation results.

Figure S10 shows the acoustic pressure for diaphragm thicknesses from 30 μm to 80 μm, driven by 0-18V sin wave. 30μm thickness diaphragm is almost 15dB better than the 80μm one.


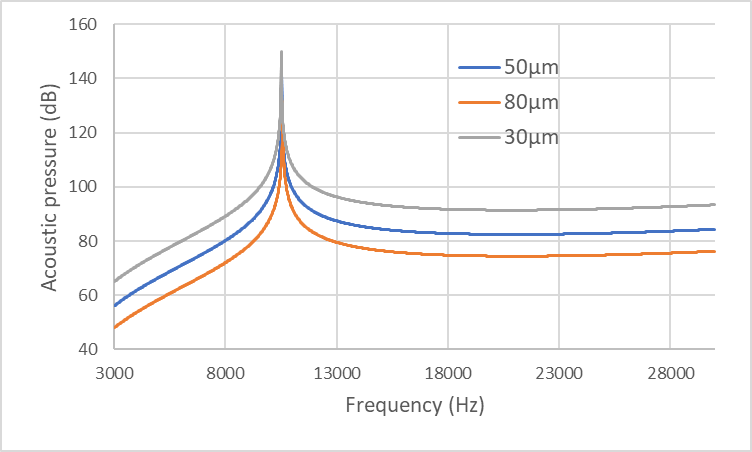


**Figure S10. Acoustic pressure of different diaphragm thicknesses with nickel pad (Simulation).**


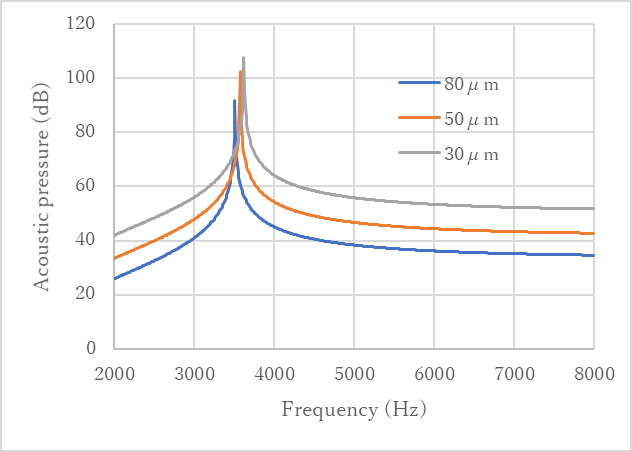


**Figure S11. Acoustic pressure simulation results with pad and silicone rubber for different diaphragm thickness cases**..

Figure S11 shows acoustic pressure simulation results with pad and silicone rubber, it is driven by 0-18 V sin wave. In this case, the Si-diaphragm thickness was changed from 30 to 80 μm. The Si-diaphragm thickness reduction is improved by 15 dB for acoustic pressure and the resonance frequency is increased by 120 Hz.

**Figure S12. Acoustic pressure simulation model with space between MEMS and horn with silicone rubber.**


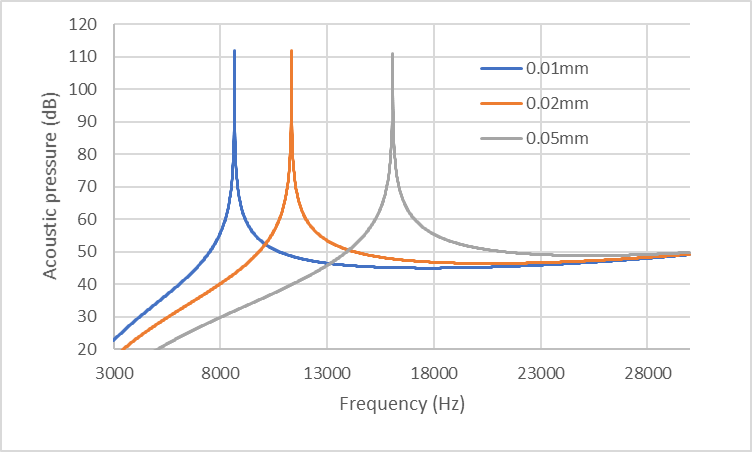


**Figure S13. Acoustic pressure simulation results of the pad with silicone rubber (space between diaphragm and horn).**

Figure S12 shows the analysis model with a small gap between the MEMS and the horn. Figure S13 shows the acoustic pressure simulation results of a metal pad with silicone rubber with a small gap, As the space increases, the resonant frequency increases.


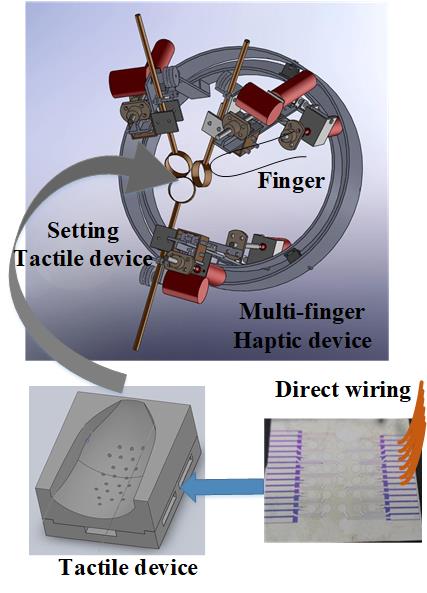


**Figure S14. Configuration of the tactile and haptic cosensation system.**

Figure S14 shows the tactile device sets the finger support for a haptic sensation system of multiple fingers.
